# Exosomal insights into ovarian cancer stem cells: revealing the molecular hubs

**DOI:** 10.1186/s13048-025-01597-3

**Published:** 2025-01-31

**Authors:** Kiana Sojoudi, Maryam Solaimani, Hossein Azizi

**Affiliations:** 1 Department of Biology, Faculty of Sciences, University of Guilan, Rasht, Iran; 2 Faculty of Biotechnology, Amol University of Special Modern Technologies, Amol, 49767 Iran

**Keywords:** Ovarian cancer, Exosome, Cytoscape, PPI network, STRING

## Abstract

Ovarian cancer is a deadly disease, often diagnosed at advanced stages due to a lack of reliable biomarkers. Exosomes, which carry a variety of molecules such as proteins, lipids, DNA, and non-coding RNAs, have recently emerged as promising tools for early cancer detection. While exosomes have been studied in various cancer types, comprehensive network analyses of exosome proteins in ovarian cancer remain limited. In this study, we used a protein-protein interaction (PPI) network. Using the Clustermaker2 app and the MCODE algorithm, we identified six significant clusters within the network, highlighting regions involved in functional pathways. A four-fold algorithmic approach, including MCC, DMNC, Degree, and EPC, identified 12 common hub genes. STRING analysis and visualization techniques provided a detailed understanding of the biological processes associated with these hub genes. Notably, 91.7% of the identified hub genes were involved in translational processes, showing an important role in protein synthesis regulation in ovarian cancer. In addition, we identified the miRNAs and LncRNAs carried by ovarian cancer exosomes. These findings highlight potential biomarkers for early detection and therapeutic targets.

## Introduction

Ovaries play a significant role in women’s reproduction system by producing eggs (ova) and hormones including estrogen and progesterone. Thus, ovaries malfunction can lead to significant health complications. Ovarian cancer (OC) is a global burden that accounts for the fifth most common cause of mortality [1]. It is aggressive in nature and often discovered at advanced stages of cancer [2]. Currently, there are 5-year survival rates for under 50% of women and 15% of women lose their lives after 2 months of being diagnosed. Moreover, it is predicted that the worldwide occurrence of ovarian cancer will increase to 371,000 cases per year by 2035 [3]. Breast or ovarian cancer in the family history of an individual is counted as the main risk factor [4]. Frequent histological types of epithelial ovarian cancer include clear cell, mucinous, serous, and endometrioid. Furthermore, it can have two subtypes: Type I and Type II tumors. Type I are less aggressive than Type II, and are detected in earlier stages. Meanwhile, Type II has a rapid progression and often contains p53 mutations [5]. Currently, the main treatments for ovarian cancer are targeted therapies, cytoreductive surgery, and platinum-based chemotherapy [6]. Even though the recurrence has been postponed, it often returns, especially in the advanced stages [7]. Ovarian cancer contains mild symptoms in its initial stages which can result in it being neglected [8]. Therefore, the detection of early diagnostic markers can play an instrumental role in patients’ survival rate and treatment. Ovarian cancer stem cells are known to be the primary source of tumor initiation and metastasis, making them a key focus of recent research [9, 10]. Among their notable features, exosomes have gained significant attention for their unique properties and critical role in intercellular communication [11, 12]. Exosomes are small vesicles (50–150 nm diameter) with lipid bilayer membranes secreted by various cells such as mesenchymal stem cells and cancer cells [13–15]. Their release is triggered by pathogenic factors or normal physiological factors [16, 17]. They are formed within multivesicular bodies (MVBs) through the inward budding of the membrane, creating small vesicles that encapsulate proteins and RNA. After MVB formation, they migrate to the plasma membrane, where they can either fuse with it to release exosomes into the extracellular space or be degraded. This fusion process is regulated by specific proteins, including Rab GTPases, which are crucial for MVB trafficking, and tetraspanins, which assist in sorting cargo into exosomes [18–21]. They contain genetics and protein material and play roles in disease onset, progression, and intercellular communication [22, 23]. They can transfer mRNA and non-coding RNAs [24]. Non-coding RNAs are broadly categorized into microRNAs (miRNAs) and long non-coding RNAs (lncRNAs) [25, 26]. miRNAs are a class of noncoding single-stranded RNAs encoded by endogenous genes, about 22 nucleotides in size. Numerous microRNAs have emerged as valuable biomarkers for various medical conditions. These include neurodegenerative disorders such as Alzheimer’s disease, metabolic diseases like diabetes, infections caused by viruses, and various types of cancer. Currently, many studies have shown that miRNAs are connected to the progression of ovarian cancer and their up or down-regulation is responsible for the prognosis of OC patients so they show a broad impact on gene expression patterns in ovarian cancer [27, 28]. Similarly, lncRNAs have vital roles including post-translation modification, differentiation, cell death, gene expression, splicing, and proliferation [29]. Moreover, lncRNAs have been associated with metastasis and tumor microenvironment in cancers such as ovarian cancer [30]. Therefore, studying their expression level can aid in cancer diagnosis and therapy.

Stem cells in ovarian cancer can be isolated via various techniques, including the fluorescent-activated cell storing method (FACS), and the Magnetic-activated cell storing method (MACS) from solid tumors [31]. Once isolated, exosomes from these ovarian cancer stem cells can be extracted through methods such as ultracentrifugation, differential centrifugation, sucrose density gradient centrifugation, commercial kits, and microfluidic techniques [32–37].

One of the key advancements in ovarian cancer diagnosis is liquid biopsy techniques which involves analyzing circulating DNA and RNA in body fluids [38]. Exosomes released by ovarian cancer stem cells play a crucial role in this context as they may enter the bloodstream, carrying information about the tumor’s status and progression, and can be used as a promising biomarker in liquid biopsy [39, 40]. There is a growing amount of research on the role of exosomal biomarkers in cancer diagnosis. However, there remains a need for further studies on the genetics and transcriptomic materials within exosomal cancer cells. Our aim in this research is to identify hub genes and non-coding RNAs in exosomes driven out of patients having ovarian cancer via bioinformatic tools.

## Methods

### Data collection

To date, 9769 proteins have been identified in the exosomes of various organisms from different diseases and cells. Proteins identified in exosomes extracted from ovarian cancer stem cells were downloaded from the ExoCarta database (http://www.exocarta.org), which includes 2035 proteins from different families. Additionally, the genes were validated through other datasets including The Human Protein Atlas (https://www.proteinatlas.org), and Microvesicles (http://microvesicles.org). These protein sets were transferred to the Cytoscape platform for further analysis. Also, miRNA and lncRNA that are identified in exosomes were downloaded from ExoCarta.

### Construction and claustration of the Protein-Protein Interaction (PPI) network

Search Tool for the Retrieval of Interacting Genes/Proteins database (STRING) is a database that can search for all known and predicted interactions between proteins, including physical interactions and functional associations; then generate the Protein-protein interaction (PPI) network consisting of all these proteins and all the interactions between them. We have created a PPI network of 2035 proteins identified in ovarian cancer cells’ exosomes using the STRING app (Version 2.0.3) in the Cytoscape platform (version 3.10.2). We used STRING: Protein query as our data source to generate the network. Edge score was calculated based on molecular action and the minimum confidence score cut-off for nodes was set at 0.400. The network was created, designed, and organized on the Cytoscape platform. Subsequently, we clustered this network using clusterMaker2 (version 2.3.3). ClusterMaker2 is a Cytoscape app that unifies different clustering, filtering, ranking, and dimensionality reduction algorithms along with appropriate visualizations into a single interface. We used Molecular Complex Detection (MCODE) network cluster algorithms. The MCODE algorithm finds highly interconnected regions in a network.

### Identification of hub proteins

In order to identify the most important proteins and score them, we used the CytoHubba plugin that is implemented in Java, based on the Cytoscape API. was used to calculate node importance in a biological network based on different criteria including Maximal Clique Centrality (MCC), Density of Maximum Neighborhood Component (DMNC), Degree, and Edge Percolated Component (EPC). By comparing the obtained data, common genes were identified and we created a separate network.

### Gene set enrichment analysis

To recognize the protein class of the identified hub genes, we loaded this gene set into PANTHER (http://www.pantherdb.org/; accessed on 14 May 2024) which is an online tool. The STRING Enrichment plugin in the Cytoscape platform was used to predict the potential functions and the biological roles of the hub genes. By using different databases, related pathways and functions were predicted with their *p*-values, and the data visualized by SRplot online tool.

### Identification of miRNAs and lncRNAs

All miRNAs and LncRNAs that are carried by exosomes were downloaded from the ExoCarta database. They were compared with the set of miRNAs and LncRNAs identified in ovarian cancer, which were extracted from mir2Disease (http://www.mir2disease.org/) [41] and LncRNADisease (http://www.rnanut.net/lncrnadisease/) [42] databases, respectively.

## Results

**Fig. 1:**
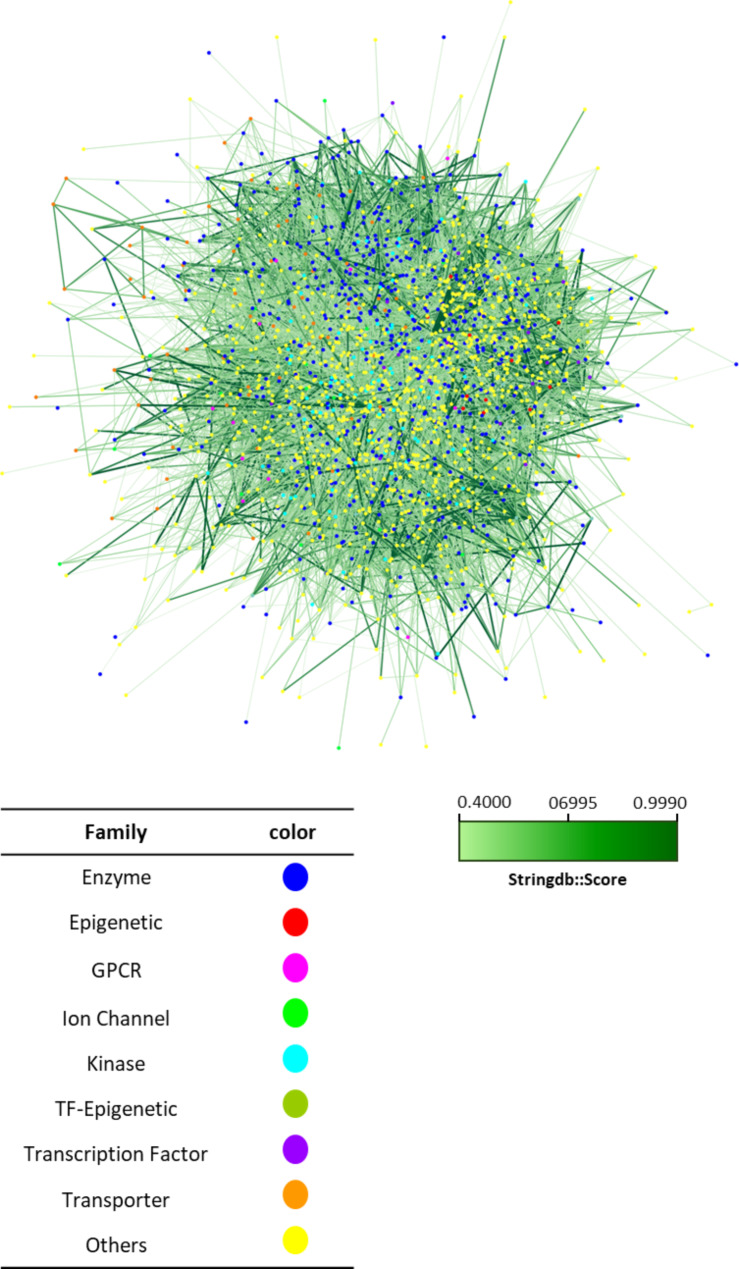
All genes carried by exosomes released from ovarian cancer cells. Identifying all the proteins that are transported by exosomes from ovarian cancer cells by using the exoCarta database. Node color represents their protein family and the degree of transparency of the edges indicates their importance according to the STRING scoring system

The STRING database was used to predict the protein-protein interaction network of exosome-carried proteins in ovarian cancer. This network, consisting of 60,750 edges, is visualized in Cytoscape—a versatile and extensible platform equipped with various plugins (Fig. 1). These plugins enhance Cytoscape’s capabilities for network research by providing new visualization tools. Researchers can easily access the graphical representation of the network and annotate the interactome with diverse types of data, including results from large-scale genome-wide studies and information about individual protein functions. Additionally, nodes within the network can be scored and ranked using data from multiple Cytoscape plugins. STRING database predicts the interactions on the basis of evidence sources such as text mining, experimental evidence, databases, co-expression, neighborhood, gene fusion and co-occurrence. The confidence score was set as > 0.400 which means PPI’s with STRING-score lower than 0.4 were not included in the study because of their low confidence score for interaction. In the network, each node stands for a protein, with all variants such as splice isoforms or post-translational modifications combined together, meaning one node encompasses all proteins emanating from a single gene locus that codes for proteins. The connections, or edges, between these nodes signify interactions between proteins. These interactions are intended to be precise and significant, suggesting that the proteins are working together towards a common function. The higher the number of connecting lines, the stronger the evidence for these protein-protein interactions. The colors of the edges are shown from bold to light based on their score. Also, we have divided the colors of nodes into 9 categories based on their families including Enzyme, Epigenetic, G Protein-Coupled Receptors (GPCRs), Ion Channel, Kinase, Transcription Factor (TF)-Epigenetic, Transcription Factor, Transporter, and others.

**Fig. 2:**
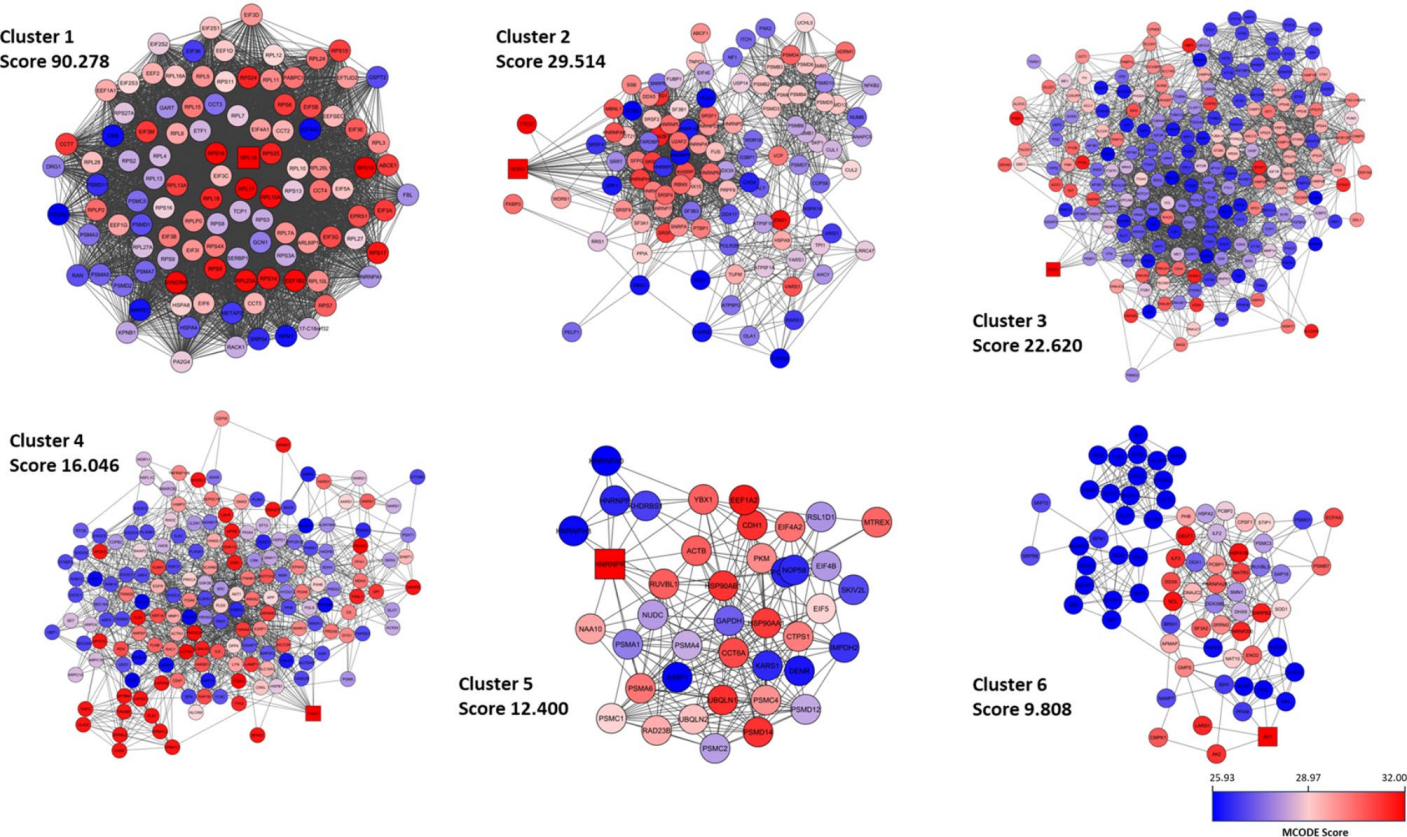
Clustering the Native network. The native network is broken down into 6 clusters up to the motif level. Each module subnetwork from the native network represents with MCODE score. Squares represent the Seed of each cluster

Using the Clustermaker2 app we have shown that there are 6 important clusters in this network (Fig. 2). Clustermaker2 provides several clustering algorithms for clustering data within columns as well as clustering nodes within a network. Clusters are highly interconnected regions in a network like protein complexes and parts of pathways. We used the Molecular Complex Detection (MCODE) which is a network partitioning algorithm. This algorithm is generally used for finding modules and complexes within protein-protein interaction networks. In a cluster, the node with the top score is known as the Seed. This is the originating node of the cluster and is depicted as a square shape.

**Fig. 3:**
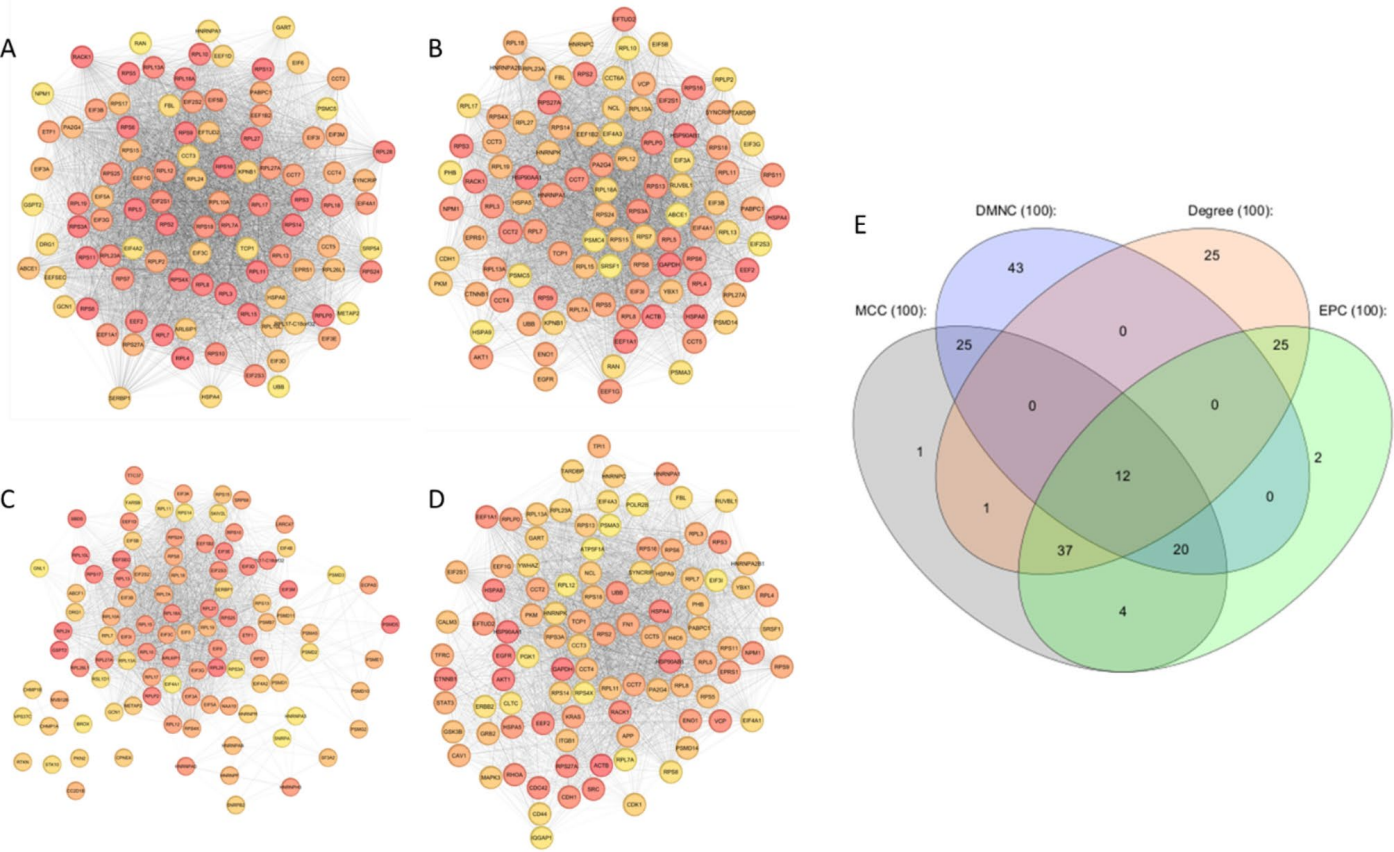
Identification of hub genes by cytoHubba plugin. CytoHubba plugin of Cytoscape predicted hub genes based on 4 different algorithms including MCC (**A**), EPC (**B**), DMNC (**C**), and Degree (**D**). The Venn diagram is applied to demonstrate the common predicted hub genes (**E**)

**Fig. 4:**
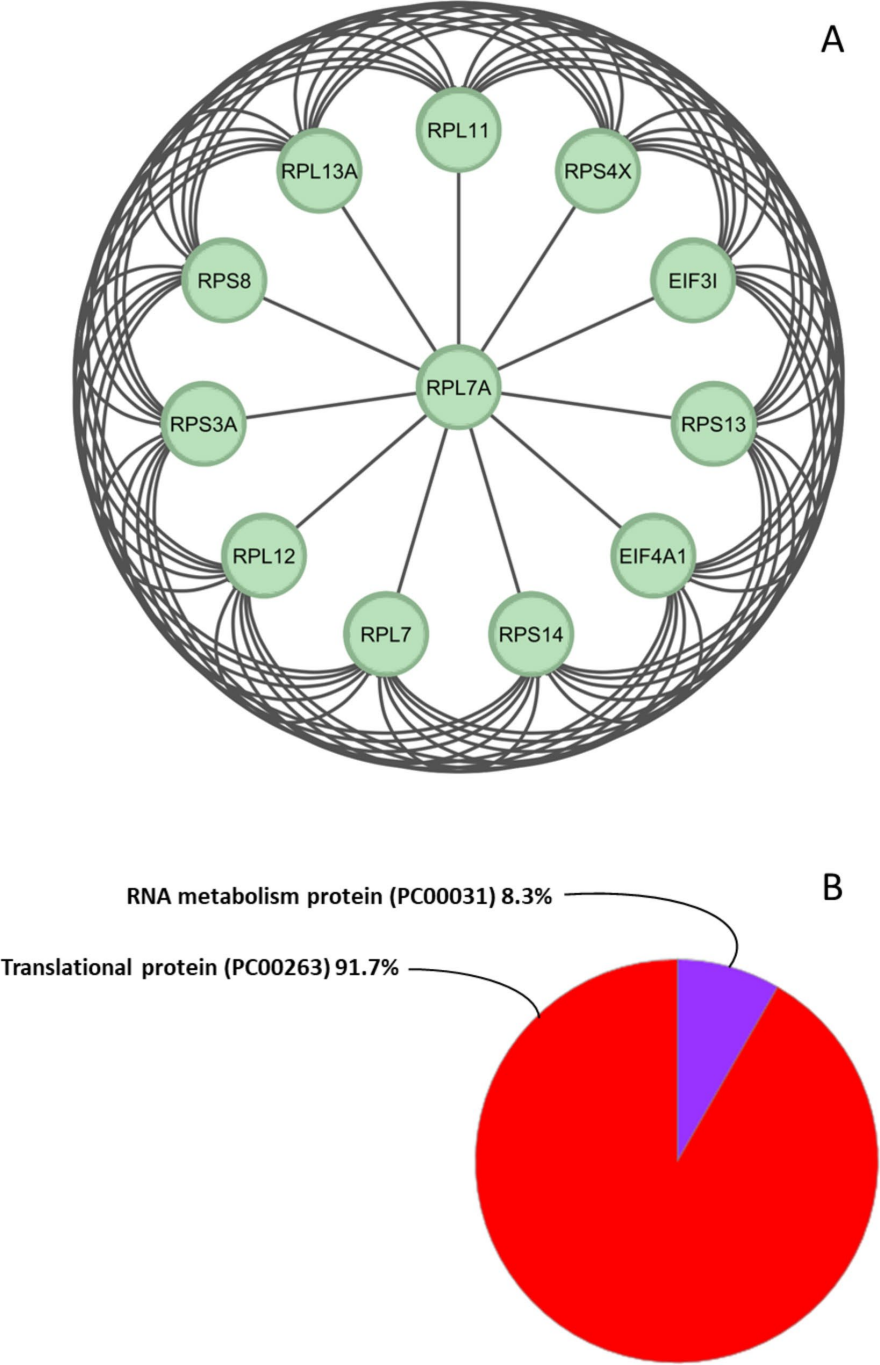
The network of common hub-genes and PANTHER analysis. Protein connections between 12 common genes identified among cytoHubba algorithms were predicted and designed as a separate network (**A**). Determining the protein classification of this network. Each color represents a special description (**B**)

**Fig. 5:**
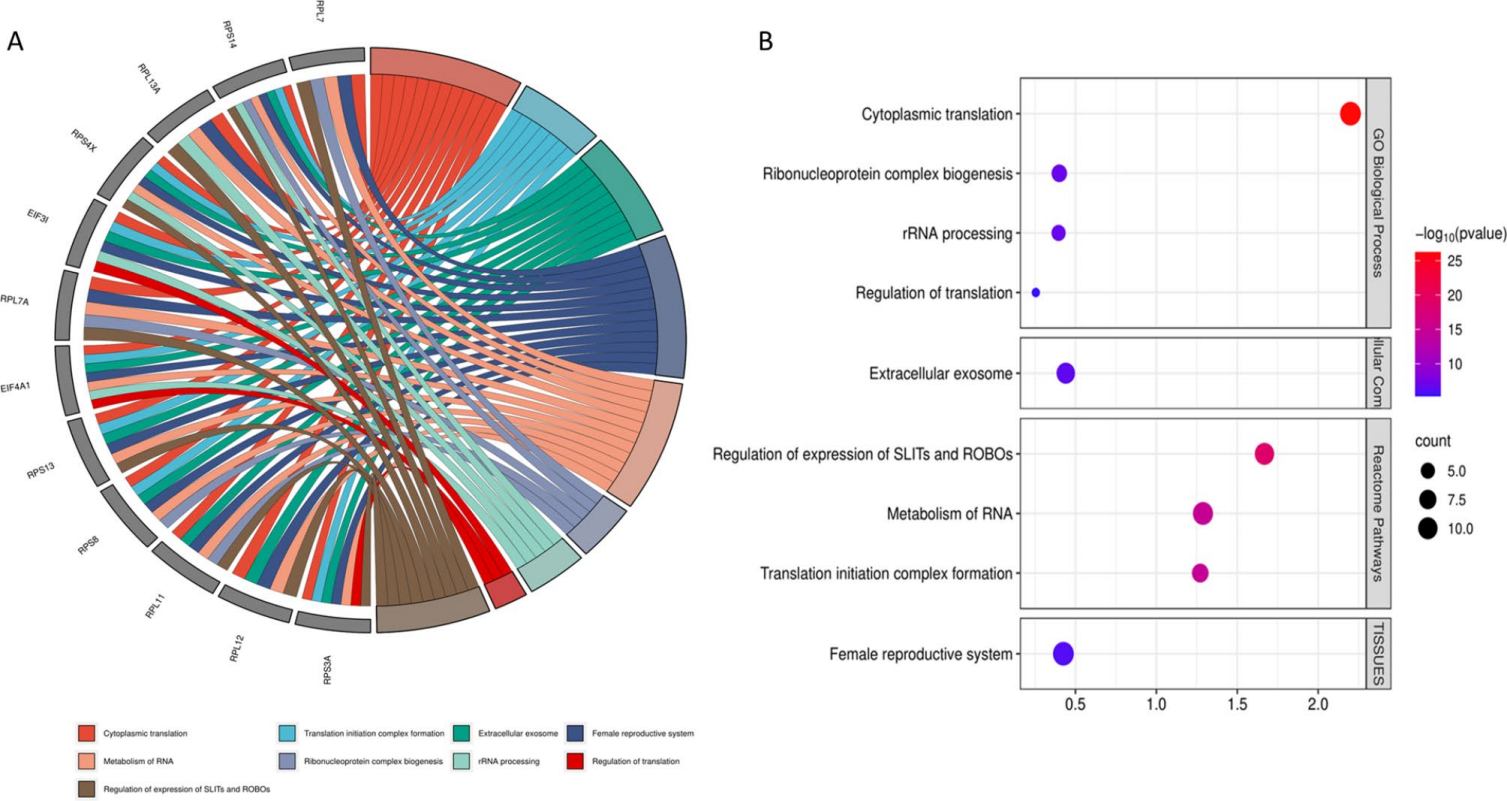
The enrichment analysis of the hub genes. The functional enrichment analysis of common hub genes by the STRING plugin in Cytoscape using 4 different databases. The chord plot demonstrates major important pathways associated with hub genes (**A**) and the Bubble plot also shows the same function with better detail including the *p*-value and the size of the bubble representing the number of genes involved in a specific term (**B**)

Subsequently, we used the CytoHubba plugin to explore important nodes and fragile motifs in the mother network by several topological algorithms (Fig. 3. A, B, C, D). The CytoHubba plugin offers an easy-to-use platform for network analysis, featuring eleven different scoring techniques. It has been developed to streamline the process for biologists to analyze networks and leverage extra network properties. With the enhanced node retrieval function in the CytoHubba control panel, researchers can navigate and investigate the network, as well as isolate subnetworks that are of particular interest to them. As a single algorithm might produce false positives, we adopted a four-fold algorithm to jointly identify hub genes including Maximal Clique Centrality (MCC) (Fig. 3. A), Density of Maximum Neighborhood Component (DMNC) (Fig. 3. C), Degree method (DEG) (Fig. 3. D), and Edge Percolated Component (EPC) (Fig. 3. B). Each node of the PPI network was colored from highly essential (red) to essential (yellow) according to the scores of the nodes. Afterward, we identified the common hub genes by the Venn and Euler Diagrams (Version 1.0.3) plugin in Cytoscape which provides a diagram view and details view for comparing our 4 groups at a time (Fig. 3. E). Then 4 algorithms were merged and 12 common nodes were identified and we created a sub-network by them which included 66 edges (Fig. 4. A). These 12 proteins were separately analyzed using the PANTHER (Protein ANalysis Through Evolutionary Relationships, Foster City, CA, USA) to identify protein classes. The results were displayed as a pie chart (Fig. 4. B). The PANTHER server showed that 91.7% of these proteins are translational proteins and 8.3% of them are RNA metabolism proteins. In addition, by STRING enrichment analysis in the Cytoscape, we have predicted enriched biological processes associated with these 12 hub genes. We used 4 different databases including some GO biological processes, TISSUES, Reactome Pathways, and GO Cellular Component. STRING enrichment analysis in the Cytoscape, we have predicted enriched biological processes associated with these 12 hub genes. We used a chord plot (Fig. 5. A) to show the relationship between terms and genes and also used a Bubble plot (Fig. 5. B) in which *p*-values are represented by colors and gene counts are represented by bubble size.

**Fig. 6:**
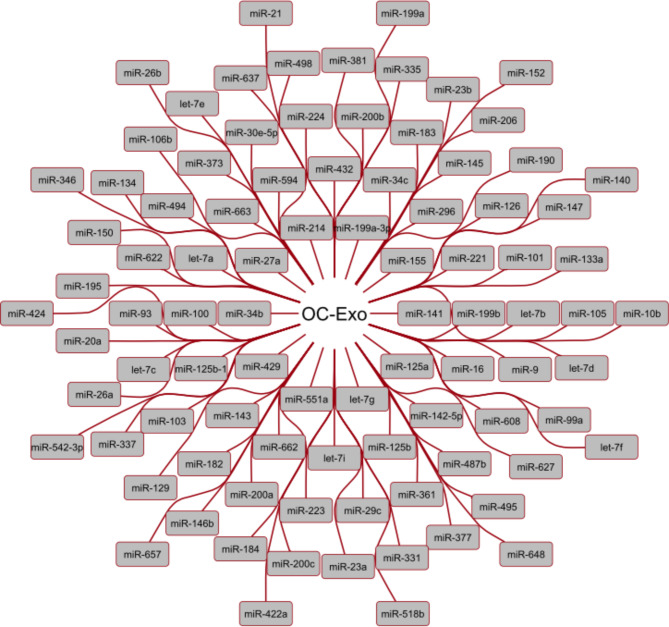
miRNAs that are carried by ovarian cancer cells’ exosomes. Overall, 91 miRNAs were identified that are carried by exosomes derived from ovarian cancer cells

**Fig. 7:**
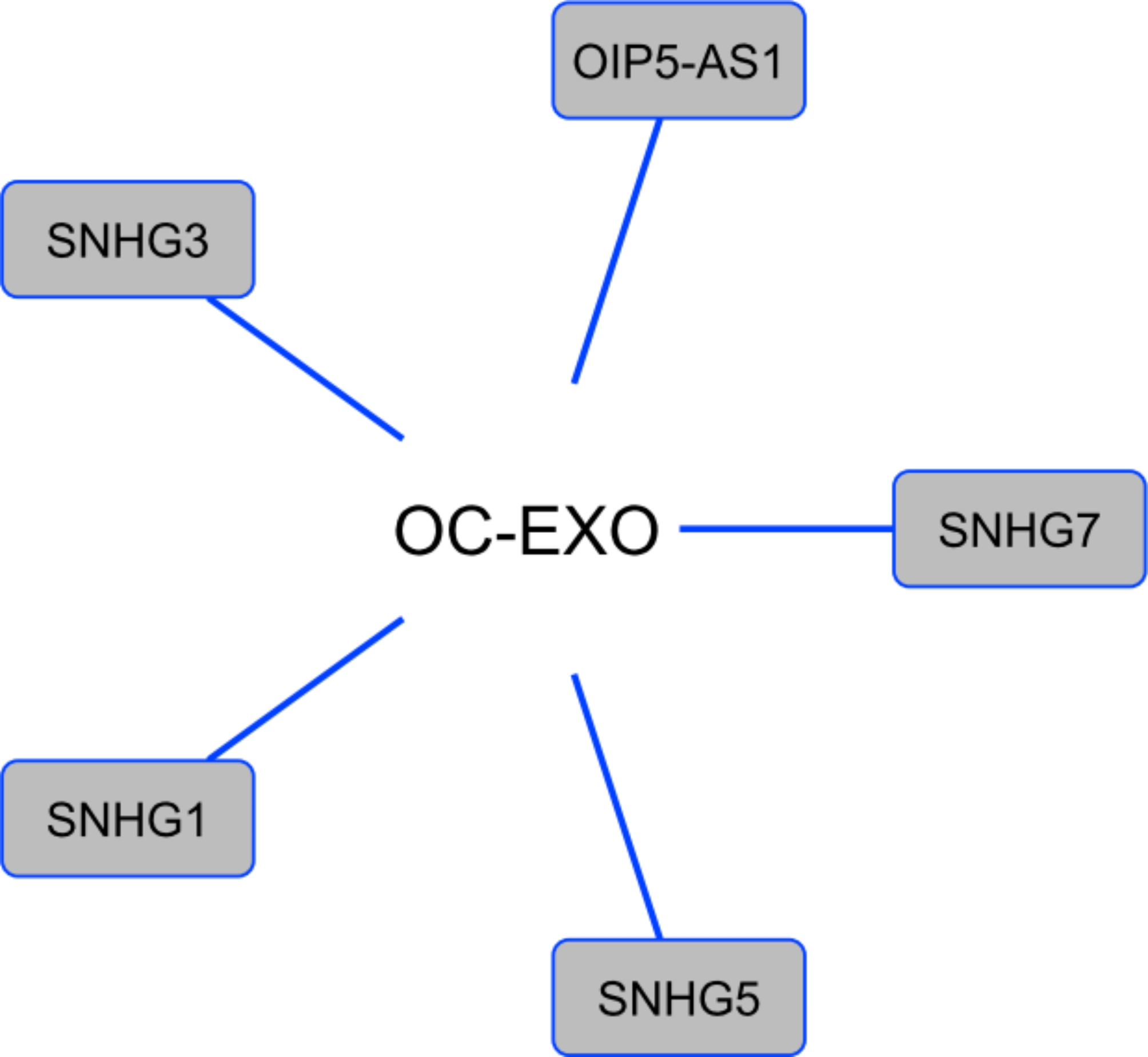
LncRNA that are carried by ovarian cancer cells’ exosomes. Overall, 5 LncRNAs were identified that are carried by exosomes derived from ovarian cancer cells

To identify the miRNAs and LncRNAs that are carried by the exosomes from ovarian cancer cells, we compare the total miRNAs and LncRNAs that are carried by exosomes in the body with the predicted miRNAs and LncRNAs that are involved in ovarian cancer. The result suggests that 91 miRNAs (Fig. 6) as well as 5 lncRNAs (Fig. 7) can carried by the exosomes extract from ovarian cancer cells.

## Discussion

Ovarian cancer is often diagnosed at advanced stages due to the absence of reliable biomarkers, which makes early detection critical for improving survival rates [43]. Therefore, detecting novel biomarkers is an urgent need for successful treatment. Exosomes have recently emerged as a promising diagnostic for early detection of cancer in ovarian cells. They contain various molecules including proteins, lipids, DNA, and non-coding RNAs including miRNAs and lncRNAs, and are secreted by most cell types [44]. It is undeniable that a thorough analysis of the protein-protein interaction (PPI) network of exosome proteins in ovarian cancer leads to a better understanding of the disease’s molecular basis. Thus, there have been various studies regarding exosomes’ role as an effective biomarker in different cancer types but a comprehensive network analysis of exosome-carried proteins specifically in ovarian cancer has been limited.

We employed a high-confidence PPI network, with a stringent confidence score threshold (> 0.400), which ensures the reliability of our interactions. Six important clusters were identified through the network via Clustermaker2 and the MCODE algorithm which highlighted regions that may present functional proteins or pathways. Using a four-fold algorithmic approach (MCC, DMNC, Degree method, and EPC), we identified 12 common hub genes including RPL11, RPS4X, EIF3I, RPS13, EIF4A1, RPS14, RPL7, RPL12, RPS3A, RPS8, RPL13A, and RPL7A. Our study is among the few to perform a comprehensive network analysis of exosome-carried proteins in ovarian cancer stem cells. This bioinformatics-driven approach identifies potential biomarkers in ovarian cancer stem cells. Our multi-algorithm approach lessens the risk of false positives, enhancing the reliability of identified hub genes. Furthermore, a detailed understanding of biological processes related to the hub genes was provided via employing STRING and visualization through chord and bubble plots. These biological processes include cytoplasmic transition, ribonucleoprotein complex biogenesis, rRNA processing, regulation of translation, extracellular exosome, regulation of expression of SLITs and ROBOs, metabolism of RNA, translation initiation complex formation, and female reproductive system. The high proportion of translational proteins (91.7%) among the identified hub genes indicates a significant role in protein synthesis regulation in ovarian cancer. As exosomes are known to carry cellular content that reflects the state of their cell of origin, and the hub-genes that we identified are primarily ribosomal proteins and translation initiation factors, the presence of these 12 hub-genes in exosomes may indicate altered ribosomal biogenesis or protein synthesis in ovarian cancer stem cells.

Similar to our findings, Ribosomal Protein L11 (RPL11) has been identified to play a role in various cancers such as esophageal squamous carcinoma, fibroblasts, colorectal cancer, and gastric cancer [45]. A study by Hsu et al. showed that RPL11 is involved in Protein translation, cell proliferation, and regulation of programmed cell death (apoptosis) [46]. RPL11 has the potential to be used as a biomarker in ovarian cancer due to its expression level and functional involvement in tumor biology. Increased levels of RPL11 have been related to better prognostic outcomes in certain cancers since they connect with the activation of p53 and subsequent tumor suppression [47]. On the other hand, decreased levels of expression can show a compromised p53 pathway, which is commonly seen in many cancers, including ovarian cancer [48, 49].

In addition to RPL11, our study highlights several other ribosomal proteins that have been implicated in cancer progression, such as Ribosomal Protein S4 X (RPS4X) and eukaryotic translation initiation factor 3 subunit i (eIF3i). RPS4X is located on the X chromosome and provides instructions for making a protein called RPS4X. This protein is a part of the 40 S subunit of the ribosome, which is a cellular structure essential for protein synthesis [50]. Research by Tsofack et al. indicated that RPS4X could be employed as a marker to identify resistance to platinum-based therapy in ovarian cancer [51]. Increased levels of RPS4X have been shown to be connected to a better overall survival rate among ovarian cancer patients. High expression of RPS4X has been associated with a decreased risk of death and disease progression, making it a promising prognostic marker. Conversely, low expression levels are connected to poorer outcomes and more aggressive disease [52]. The expression levels of RPS4X could inform treatment decisions by offering insights into how patients may react to cisplatin-based therapies, thereby aiding in the personalization of treatment strategies [51, 53]. This highlights the biomarker’s potential utility in clinical settings.

Similar to our results, research by Zhang et al., reports that a specific protein called eIF3i plays a role in promoting tumor growth by controlling the translation of certain genes. It also states that levels of eIF3i are elevated in colorectal cancer (CRC) and that this protein supports the growth of CRC cells [54]. It has been shown that eIF3i expression level changes significantly between cancer tissues and surrounding normal tissues. For instance, it has been indicated that the changed expression of translation initiation factors, including eIF3i, is associated with cancer cell survival and metastasis [55]. Increased levels of eIF3i expression have been connected with worse prognostic results in patients with ovarian cancer. Studies show that eIF3i could aid as a prognostic marker for understanding tumor progression and patient prognosis [56–58].

Our findings also align with study of Liu et al., which identifies YTH domain-containing family protein 1 (YTHDF1) as a key ‘reader’ of N6-methyladenosine (m6A) that targets EIF3C, enhancing overall protein production in cancer cells. YTHDF1 amplification is common in ovarian cancer and its upregulation is related to poor prognosis. The findings suggest that targeting the YTHDF1-EIF3C axis could be a valuable therapeutic strategy for treating ovarian cancer [59].

Moreover, studies using artificial intelligence have identified genes like Ubiquitin carboxyl-terminal hydrolase 19 (USP19) and RPL23 as crucial for predicting cancer recurrence. Lower USP19 levels and higher Ribosomal protein L23 (RPL23) levels were linked to poorer prognoses [60].

Zhao et al. indicated that Programmed cell death protein 4 (PDCD4) is a tumor suppressor gene that hinders cancer cell growth and metastasis by interacting with eIF4A, highlighting its potential as a diagnostic marker [61], which is similar to our findings. Studies have demonstrated that overexpression of PDCD4 leads to cell cycle arrest by upregulating inhibitors such as p21 and p27, eventually suppressing tumor growth. The biological effects of PDCD4, as well as its involvement in apoptosis and protein translation regulation, further highlight its significance in cancer biology. Given its tumor-suppressive role, PDCD4 is a valuable biomarker and a potential therapeutic target [62, 63].

Ribosomal protein S14 (RPS14), is another key protein from our findings. Tumor-suppressing effects of RPS14 have been studied in previous research. A study by Xu et al. demonstrated that RPS14 and RPL10, play a role in suppressing tumors, and RPS14 has been shown to activate Transcriptionally Active p73 (TAp73), which is similar to the p53 protein, and to promote apoptosis [64]. Additionally, RPS14 can inhibit the activity of cancer-promoting proteins like c-Myc [65, 66]. Targeting RPS14 or its related pathways could provide novel strategies for treatment [64]. Research into RPS14-targeted therapies could offer new insights for enhancing treatment outcomes in ovarian cancer patients.

Our results demonstrated Ribosomal protein L7 (RPL7), as a hub gene in exosomes driven from ovarian cancer stem cells. Similar to our findings, Alexandrova et al. showed that Ribosomal protein L7 (RPL7) and RPL7A are associated with EIF2 Signaling in ovarian cancer [67]. In consistency with our results, a study by Li et al. compared high-grade serous ovarian cancer (HGSOC) samples at different stages, and certain genes including Ribosomal protein L12 (RPL12) were found to be involved in different biological processes [68].

Butyrophilin subfamily 3 member A3 (BTN3A3) acts as a tumor suppressor in various cancer types including clear cell renal cell carcinoma (ccRCC). The study by Li et al. reveals that BTN3A3 inhibits the proliferation, migration, and invasion of ccRCC cells by binding directly to Ribosomal Protein S3A (RPS3A). Overexpression of both BTN3A3 and RPS3A increases cellular oxygen consumption rate (OCR) and reactive oxygen species (ROS) levels, which play a role in regulating the Mitogen-activated protein kinases (MAPKs) pathway and tumor cell growth [69]. Research shows that BTN3A3 expression is significantly lower in ovarian cancer tissues compared to normal ovarian tissues. Specifically, elevated expression levels of BTN3A3 have been connected with better patient survival rates, while decreased expression connects with a more hostile tumor phenotype. Studies using various ovarian cancer cell lines have demonstrated that knocking down BTN3A3 increases the proliferation, migration, and invasion capabilities of these cancer cells [70, 71]. These studies align with our findings regarding this protein, highlighting its potential role as a protective molecular marker.

Research conducted by Chen et al. indicated that Proline and arginine-rich end leucine-rich repeat protein (PRELP), Galectin 1 (LGALS1), and Ribosomal protein S8 (RPS8) could play important roles in predicting the prognosis of pancreatic cancer. In particular, Ribosomal protein S8 (RPS8) and LGALS1 have the potential to be targeted for therapy in order to enhance patient survival [72]. RPS8, like other ribosomal proteins, is involved in protein synthesis and ribosome biogenesis, processes important for cell growth and metabolism. Its dysregulation can lead to abnormal protein translation, often observed in cancer cells. RPS8 has been implicated in various signaling pathways that increase tumorigenesis [73]. In consistency with our results, this protein shows potential as not only a biomarker but also a target for therapeutic interventions.

In research by Bian et al., it is stated that Ribosomal protein L13a (RPL13A) is a good reference gene to use when adjusting the expression levels of genes in ovarian cancer cells after they have been treated with anti-cancer drugs such as paclitaxel and 10-hydroxy camptothecin [74]. This aligns with our findings, and its usage as a biomarker. RPL13A plays essential roles in protein synthesis and ribosome biogenesis [75]. Dysregulation including RPL13A, can lead to altered protein synthesis, often associated with cancer progression. Its functional involvement in cellular processes like stress responses and apoptosis may further highlight its relevance in the context of malignancies, including ovarian cancer [76].

Furthermore, in our study, 91 miRNAs and 5 LncRNAs have been identified to be carried by exosomes driven from ovarian cancer stem cells. Notable miRNAs include miR-21 and members of the let-7 family, both of which have been previously implicated in cancer progression and therapeutic resistance [77, 78]. Similarly, we found lncRNAs, including Small Nucleolar RNA Host Gene 3 (SNHG3) and Small Nucleolar RNA Host Gene 7 (SNHG7), to be enriched in ovarian cancer stem cell exosomes, highlighting a potential role in stem cell-like properties and tumor aggressiveness. These findings align with prior studies that emphasize the role of exosomal miRNAs and lncRNAs in cancer pathways.

For instance, previous studies consistently showed high expression of miR-21 in ovarian cancer tissues compared to normal ovarian tissue. miR-21 targets the tumor suppressor gene Phosphatase and Tensin Homolog (PTEN) and leads to the activation of the PI3K/Akt pathway which promotes cell proliferation and survival [79, 80]. A study by Yeung et al. shows that exosomal miR-21 gets transferred from the neighboring stromal cells, and is associated with aggressive types of ovarian cancer and resistance to chemotherapy [81]. Research by Koutsaki et al. demonstrates that exosomal miR-200a, miR-200b, and miR-200c can effectively differentiate between women with epithelial ovarian cancer and healthy women, as well as differentiate between malignant (cancerous) and benign (non-cancerous) ovarian tumors, which further highlights their potential use as biomarkers [82]. In ovarian cancer, exosomal let-7 acts primarily as a tumor suppressor microRNA [83]. Let-7 can target several oncogenes, including RAS, HMGA, and c-Myc, and studies have shown that higher levels of let-7 within exosomes from highly invasive ovarian cancers may be associated with a more aggressive tumor phenotype [84, 85].

Among lncRNAs, we found SNHG3 and SNHG7 to be enriched in ovarian cancer stem cell exosomes. Similar to our findings, a study by Huang et al. showed the significance of exosomal SNHG3 in colorectal cancer, which leads to augmented proliferation and metastasis [86]. Additionally, research by Cui et al. showed that exosomal lncRNA SHNG7 can increase the development of new blood vessels (angiogenesis), and cell proliferation in High-Grade Serous Ovarian Cancer [87]. Certain exosomal RNAs, such as OIP5 Antisense RNA 1 (OIP5-AS1), have been studied in other cancer types but are not well-documented in ovarian cancer. This study uniquely highlights their enrichment within exosomes driven from cancer stem cells, increases our understanding of how ovarian cancer stem cells modulate the tumor microenvironment, and opens new avenues for therapeutic intervention. While our study provides valuable insights into the molecular landscape of exosome-carried proteins and RNAs in ovarian cancer, further experimental validation is necessary to confirm their functional roles. Future studies focusing on clinical samples will be critical to translating these bioinformatics findings into practical diagnostic and therapeutic applications. It is crucial to acknowledge that ovarian cancer and the detection of potential markers in its diagnosis are dynamic fields in need of regular updates. This study covers information on hub genes in exosomes and their association with ovarian cancer at a specific point in time.

## Conclusion

Early detection of ovarian cancer remains a challenge due to the lack of reliable biomarkers. This study demonstrates the potential exosome diagnostic tools. Through a comprehensive protein-protein interaction (PPI) network analysis, six significant clusters were identified. Our methodological approach, incorporating MCC, DMNC, Degree method, and EPC algorithms, allowed us to discover 12 common hub genes, mostly involved in protein synthesis regulation. The high proportion of translational proteins among these hub genes shows their potential role in ovarian cancer pathology. Several hub genes, such as RPL11, RPS4X, EIF3I, and RPS14, have been previously linked to various cancers and were confirmed to play crucial roles in ovarian cancer through this study.

## Data Availability

No datasets were generated or analysed during the current study.
